# Photobiomodulation as a Potential Treatment for Alzheimer’s Disease: A Review Paper

**DOI:** 10.3390/brainsci14111064

**Published:** 2024-10-26

**Authors:** Miaomiao Wang, Deeba Dinarvand, Clement T. Y. Chan, Anatol Bragin, Lin Li

**Affiliations:** 1 Department of Biomedical Engineering, University of North Texas, Denton, TX 76207, USA; miaomiaowang@my.unt.edu (M.W.); deebadinarvand@my.unt.edu (D.D.); tszyanclement.chan@unt.edu (C.T.Y.C.); 2 Department of Neurology, University of California Los Angeles, Los Angeles, CA 90095, USA; abragin@mednet.ucla.edu; 3 Brain Research Institute, David Geffen School of Medicine at UCLA, Los Angeles, CA 90095, USA

**Keywords:** Alzheimer’s disease (AD), photobiomodulation (PBM), non-invasive treatment

## Abstract

Background: Alzheimer’s disease (AD), the most prevalent form of dementia, is a leading neurodegenerative disorder currently affecting approximately 55 million individuals globally, a number projected to escalate to 139 million by 2050. Despite extensive research spanning several decades, the cure for AD remains at a developing stage. The only existing therapeutic options are limited to symptom management, and are often accompanied by adverse side effects. The pathological features of AD, including the accumulation of beta-amyloid plaques and tau protein tangles, result in progressive neuronal death, synaptic loss, and brain atrophy, leading to significant cognitive decline and a marked reduction in quality of life. Objective: In light of the shortcomings of existing pharmacological interventions, this review explores the potential of photobiomodulation (PBM) as a non-invasive therapeutic option for AD. PBM employs infrared light to facilitate cellular repair and regeneration, focusing on addressing the disease’s underlying biomechanical mechanisms. Method: This paper presents a comprehensive introduction to the mechanisms of PBM and an analysis of preclinical studies evaluating its impact on cellular health, cognitive function, and disease progression in AD.The review provides a comprehensive overview of the various wavelengths and application methods, evaluating their efficacy in mitigating AD-related symptoms. Conclusions: The findings underscore the significant potential of PBM as a safe and effective alternative treatment for Alzheimer’s disease, emphasizing the necessity for further research and clinical trials to establish its therapeutic efficacy conclusively.

## 1. Introduction

Alzheimer’s disease (AD) is the most prevalent form of dementia and a major neurodegenerative disorder affecting approximately fifty-five million individuals globally. This figure is anticipated to increase considerably, with projections indicating that 139 million individuals will be affected by 2050. Currently, Alzheimer’s disease is the fifth-leading cause of mortality among individuals aged 65 and above [1,2,3,4,5]. As Alzheimer’s disease is age-related, the number of diagnosed patients is expected to rise in parallel with the growth of the aging population [6]. The global prevalence of AD is projected to double every five years [7].

AD is a progressive neurodegenerative disease that initially manifests as mild memory loss, and subsequently leads to declines in language-related, cognitive, and behavioral functions [6,7,8,9,10,11].In many cases, the hippocampus and the cortex are the initial regions of the brain affected by AD [12]. This is why memory deficits and impairments in executive function are typically the initial indications of Alzheimer’s disease. Additionally, early symptoms of AD may manifest as alterations in personality, including mood swings, anxiety, and depression. As the disease progresses, it significantly impairs language, reasoning, and behavior, ultimately resulting in a complete loss of independence [13]. As the disease progresses, AD causes irreversible damage to various brain regions, including neuronal death, synaptic loss, and brain atrophy [8,9]. The AD brain exhibits three principal structural alterations. Firstly, there is an increase in the number of beta-amyloid plaques between neurons, which disrupts communication. Secondly, the aggregation of tau proteins within cells forms tangles and results in a disruption to the transport system. Lastly, the deaths of neurons and loss of synapses result in the breakdown of the neuronal communication network and a reduction in cognitive processes [2,3,6,8,9,10,11,14].

A comprehensive understanding of the pathological features of AD, including the formation of amyloid plaques and tau tangles, is essential for the development of effective treatments and strategies. Amyloid precursor protein (APP), a type 1 transmembrane protein found in numerous cells, including neurons, has been shown to play a role in neural growth and maturation [12,15]. APP is frequently subjected to processing, resulting in the formation of smaller fragments. One such fragment is the beta-amyloid protein. The aggregation of beta-amyloid plaques in the brain is toxic, resulting in axonal defects, synaptic damage, and neuronal apoptosis [12,16,17]. In a healthy brain, these plaques are cleared by microglia, which are the brain’s resident macrophages. However, in Alzheimer’s disease, glial cells malfunction, contributing to the accumulation of beta-amyloid plaques in the extracellular space [8].

Tau, a microtubule-associated protein, is indispensable for the maintenance of microtubule structure and stability in the brain [18,19,20]. By binding to the microtubules, Tau facilitates the appropriate transport of nutrients through the neuron. In patients diagnosed with AD, tau proteins have been observed to detach from microtubules and undergo aggregation, resulting in the disassembly of microtubules within the affected brain region [21]. The collapse of the microtubular structure and the formation of tau tangles result in the disruption of the neuron’s transport system, which in turn impairs synaptic communication between neurons [18].

Despite the widespread prevalence of AD and decades of research, this disease currently has no definitive cures or treatments [3,6,7]. Currently available treatments, such as the prescription drug aducanumab, are designed to slow the progression of dementia by removing beta-amyloid from the brain. However, these drugs do not constitute a cure for the disease, and there have been concerns raised about their efficacy [3]. Other medications, including donepezil and galantamine, are commonly prescribed to manage symptoms related to memory loss and cognition deficits. It is unfortunate that these medications frequently have adverse effects, including nausea, headaches, and microhemorrhages, and have demonstrated only limited efficacy [3,6,7]. The disease process of AD commences years before the onset of clinical symptoms, with a lengthy preclinical phase marked by gradual and progressive pathological alterations [3]. This highlights the critical importance of early detection and intervention. In light of the high prevalence of AD and the shortcomings of current treatments, there is an urgent need for further research and the development of alternative therapies.

One promising area of exploration for an alternative treatment is photobiomodulation (PBM). PBM is a potential non-invasive treatment for Alzheimer’s disease that utilizes infrared light to target cells, enhancing their capacities for repair and regeneration [5,7,9,10,11,22,23]. This technique has demonstrated efficacy in promoting cellular health without the adverse effects commonly associated with pharmacological treatments [6]. The safety and non-invasive nature of PBM render it an attractive proposition for further investigation. An efficacious treatment for AD not only manages the disease but also targets its underlying biomechanical mechanism [10,22].

The following section assesses the potential of PBM as a non-invasive treatment for Alzheimer’s disease. The objective of this review is to provide a comprehensive understanding of the mechanism and feasibility of PBM as an alternative treatment for Alzheimer’s disease by analyzing various pre-clinical experiments conducted on the effect of PBM on AD progression. This review paper encompasses a comprehensive range of studies on PBM in preclinical trials that explore its impact on Alzheimer’s disease. The review encompasses an array of wavelengths and application methods of PBM, with a comparative analysis of their impact on cellular health, cognitive function, and disease progression.

## 2. Mechanism of Action of Photobiomodulation

**Figure 1:**
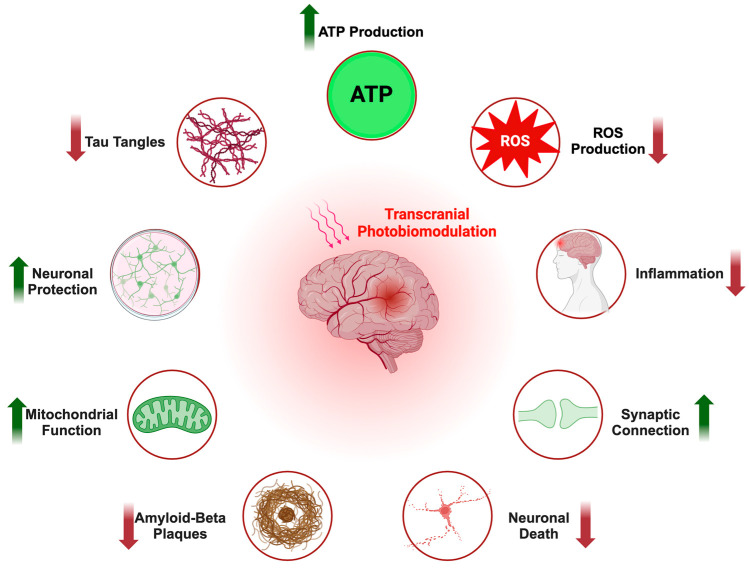
Mechanisms of PBM. This schematic shows the effects of PBM on key pathological features of AD. PBM enhances ATP production, which can improve neuronal energy balance. PBM is also shown to reduce the formation of tau tangles, amyloid-beta plaques, reactive oxygen species (ROS), and inflammation, all contributors to neurodegeneration. Furthermore, PBM enhances mitochondrial function to reduce neuronal death. Red arrows pointing downwards indicate a decrease and green arrows pointing upwards indicate an increase. This figure was created with BioRender.com (accessed on 25 October 2024).

PBM is an emerging technique that employs light to facilitate tissue growth and healing and has been demonstrated to be effective in a number of studies [5,10,11,22,23]. This approach typically delivers red or near-infrared light at low intensities to stimulate the photoreceptors, enhancing ATP synthesis, which in turn increases cell viability and metabolism [10,11,22,24]. Furthermore, PBM has been demonstrated to diminish oxidative stress, inflammation, and toxin accumulation by stimulating glial cells, thus conferring additional therapeutic advantages [7,9]. PBM has been explored as a potential therapeutic intervention for a range of neurological conditions, including traumatic brain injury, Parkinson’s disease, and Alzheimer’s disease. Although not yet a standardized treatment, PBM holds promise as a non-invasive and effective therapy that avoids many of the biological side effects associated with conventional treatments [10,24]. Recent studies have indicated that PBM can facilitate neuronal function, thereby establishing it as a promising area of research for the treatment of neurodegenerative diseases such as Alzheimer’s disease. The following section will examine the biological mechanisms that underpin PBM. A diagrammatic representation of the general mechanisms of action of PBM is provided in Figure 1.

### 2.1. Light Absorption by Chromophores

Cytochrome c oxidase (CCO) is a protein complex located within the mitochondria and represents the final enzyme in the respiratory transport chain system [5,22]. In accordance with the first law of photobiology, photons of light must be absorbed by a chromophore in the tissue in order to exert a biological effect [10,22]. In the human body, these chromophores are located inside the mitochondria. CCO is said to be the primary photo-acceptor in the mitochondria [10,22,25]. CCO functions as the final enzyme (complex IV) in the electron transport chain, which is located in the inner mitochondrial membrane [26]. This chain conducts a series of redox reactions that facilitate the movement of electrons across the inner mitochondrial membrane, ultimately creating a proton gradient. This gradient powers ATP synthase (complex V), which synthesizes ATP from adenosine diphosphate (ADP), providing essential energy for cellular processes. CCO plays a pivotal role in the electron transfer process, facilitating the transfer of electrons from cytochrome c to molecular oxygen [27]. CCO is composed of thirteen distinct polypeptide subunits, in addition to two heme groups and two copper centers, which can exist in either oxidized or reduced states, resulting in a total of sixteen possible oxidation states. These diverse oxidation states are associated with distinct absorption spectra, with CCO exhibiting a distinctive capacity for absorption in the near-infrared region, a phenomenon that is uncommon among biological molecules.

In vitro studies have demonstrated the activation of CCO by a 633 nm red laser [22,28]. An increase in intracellular ATP is one of the most frequent and significant findings after PBM, both in vitro and in vivo. CCO is a key enzyme in the mitochondrial electron transport chain, facilitating ATP production by transferring electrons to oxygen; this is sufficient to indicate the main role of CCO in the PBM experiment [29].

Investigations have also revealed that the efficacies of different wavelengths of PBM correspond closely to the absorption spectrum of CCO, particularly in the red-to-near-infrared range [22,30]. As a critical enzyme within the mitochondrial electron transport chain, CCO facilitates the transfer of electrons to oxygen, which is the final step in ATP production. By absorbing light at these specific wavelengths, CCO undergoes photo stimulation that can lead to enhanced cellular respiration and increased ATP synthesis. One significant mechanism by which light influences CCO involves the photodissociation of nitric oxide (NO), a potent inhibitor of CCO [10,11,22]. NO can bind to CCO and obstruct electron flow within the mitochondrial electron transport chain; however, when CCO is exposed to light, particularly within the red and near-infrared wavelengths, the NO is dissociated, thus reactivating CCO’s enzymatic activity [31]. This reactivation enables the mitochondria to resume efficient ATP production, which is essential for various cellular functions and energy demands [6,10,11,22].

Furthermore, the activation of CCO by PBM not only boosts mitochondrial respiration but also triggers downstream signaling pathways that contribute to enhanced cellular repair and regeneration, assisting in the repair and and regeneration of cells, and making PBM a promising therapeutic approach for a variety of conditions involving cellular dysfunction [24,30].

However, some researchers have challenged the assumptions regarding CCO. Lima et al. [32] used cell lines lacking CCO, including a *Cox10* knockout mouse and a human cell line with a mitochondrial DNA mutation affecting CCO subunits. Despite the absence of functional CCO, PBM (660 nm) still enhanced cell proliferation in both CCO-deficient and control cells. These findings indicate that while PBM induces metabolic changes, its cell proliferation-enhancing effect does not rely on CCO.

### 2.2. Mitochondrial Activation

Dysfunctional mitochondria play a significant role in the progression of AD by reducing ATP production and increasing reactive oxygen species (ROS) generation [10,33,34]. Mitochondria are major sources of ROS in mammalian cells, and in AD, dysfunctional mitochondria lead to elevated ROS levels through a process known as ROS-induced ROS release (RIRR) [10,22,35]. This feedback loop serves to exacerbate oxidative stress, which represents a pivotal factor in AD. This, in turn, results in damage to DNA, proteins, and lipids, and ultimately contributes to cellular aging and apoptosis [22].

Restoring normal mitochondrial function represents a potential strategy for neuroprotection and neurorehabilitation [6,10,11,22,25]. PBM is a promising therapy that targets mitochondria, modulating cellular functions at a molecular level [10,22]. The primary hypothesis is that PBM enhances mitochondrial activity by increasing ATP production, thereby promoting cell regeneration and repair. It has been demonstrated that PBM is capable of modulating ROS levels, a finding which is of great significance for the mitigation of oxidative stress associated with AD.

In the presence of elevated oxidative stress, ROS levels can facilitate the opening of mitochondrial channels, including the mitochondrial permeability transition pore (MPT) and the mitochondrial inner membrane anion channel (IMAC) [36]. This results in a collapse of the mitochondrial membrane potential and the subsequent generation of further ROS. A surge in ROS can result in the flooding of the cytosol, thereby activating RIRR in adjacent mitochondria and initiating a damaging feedback loop that exacerbates cellular damage in the affected region [22]. A comprehensive understanding of the biological mechanisms underlying PBM is crucial for advancing this treatment, enhancing its effectiveness, and reducing potential risks.

PBM enhances ATP generation in the electron transport system by photodissociation of NO and increasing mitochondrial membrane potential (MMP) [10,22]. An increase in MMP results in a transient surge in reactive oxygen species (ROS) production, which in turn activates the nuclear factor-κB (NF-κB) pathway. NF-κB is a principal transcription factor that regulates gene expression and plays a role in the activation of inflammatory processes [10,11,22,23]. ROS molecules possess the capacity to either activate or deactivate this pathway, depending on the circumstances [21]. The released NF-κB is transported to the nucleus, where it induces mitochondrial dynamics and the expression of genes involved in inflammation. In studies examining the effects of PBM in pathological states, the treatment has been demonstrated to reduce pro-inflammatory NF-κB activity and ROS production [10,11]. Consequently, PBM has the potential to mitigate oxidative stress and inflammation, which are two significant pathological factors in AD.

### 2.3. Modulation of Cellular Signaling Pathways

The efficacy of PBM is contingent upon the presence of mitochondria, which are integral to the restoration process. Consequently, PBM is most effective in cells that possess a substantial number of mitochondria and robust light-responsive metabolic pathways, such as neurons. PBM has been demonstrated to elicit the activation of diverse signaling pathways that facilitate neurorestoration and neuroprotection in AD. One of these pathways is the AKT/GSK3β/β-catenin pathway [22]. By emitting the near-infrared (NIR) light, protein kinase B (AKT) is activated. AKT then interacts with glycogen synthase kinase 3β, commonly referred to as GSK3β, and inhibits its activity. GSK3β is a kinase involved in cellular death and in the development of Alzheimer’s disease. This serine/threonine kinase additionally assists the hyperphosphorylation of tau proteins into tangles. Therefore, the deactivation of GSK3β by PBM-induced AKT results in the reduction of cellular death associated with AD and the promotion of neuronal survival.

Zhang at el. [37] employed a real-time single-cell analysis to reveal that LPLI (low-power laser irradiation) prevents staurosporine (STS)-induced apoptosis by inactivating the GSK-3β/Bax pathway. LPLI was shown to inhibit the activation of GSK-3β, Bax, and caspase-3, which are triggered by STS. Their findings suggest that LPLI protects against STS-induced apoptosis by disrupting the Bax translocation process via the PI3K/Akt/GSK-3β pathway, making it a potential therapy for neurodegenerative diseases linked to GSK-3β activity.

Another pathway that is subject to modulation by PBM is the level of brain-derived neurotrophic factor (BDNF). BDNF is a protein involved in the growth, protection, and survival of neuron cells in the central nervous system. Additionally, BDNF promotes synaptic connection and prevents dendritic atrophy. Studies have shown that PBM increases BDNF levels through activation of ERK and cAMP/PKA pathways [11,38,39,40]. The activation of this pathway by PBM has also been shown to result in a reduction in beta-amyloid load. The triggered cAMP/PKA/AIRT1 pathway leads to the hydrolysis of APP and increases the levels of beta-amyloid-degrading enzyme [5,41]. In this manner, PBM is capable of modulating the primary pathological factors associated with AD and enhancing neuroprotection in patients.

## 3. Current Evidence of PBM for Curing AD

**Figure 2:**
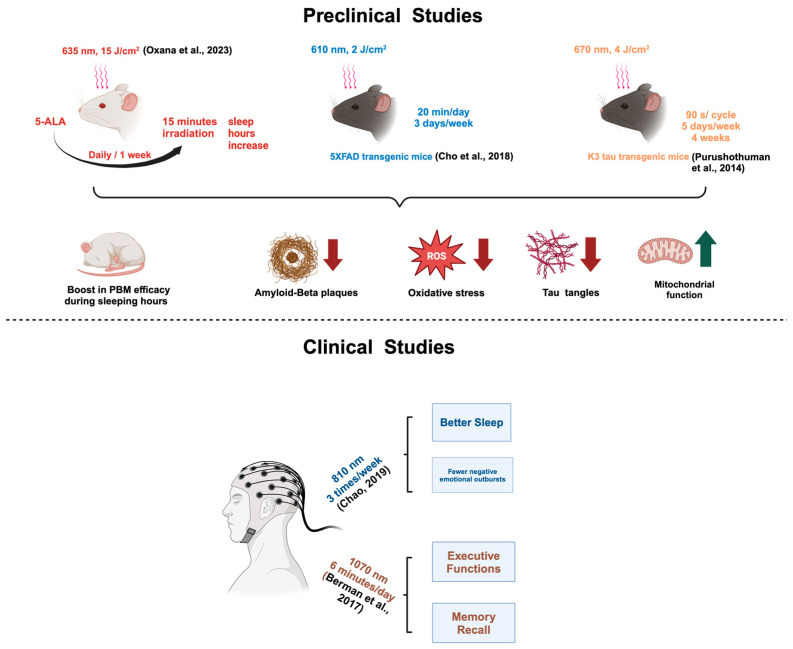
Summary of current pre-clinical and clinical studies of PBM therapy (*adapted from*). Red arrows pointing downwards indicate a decrease and green arrows pointing upwards indicate an increase. This figure was created with BioRender.com (accessed on 25 October 2024) [7,9,13,42,43].

Over the past few decades, clinical and preclinical in vivo studies have investigated the therapeutic effects of PBM in AD. A diagram illustrating these studies is presented in Figure 2.

### 3.1. Preclinical Studies of PBM for Curing AD

Preclinical studies have shown promising results, including neural protection, reduction in beta-amyloid plaques, and improvements in cognitive function. These studies predominantly utilized light wavelengths between 650–1200 nm, aligning with the absorption range of CCO in the mitochondrial electron chain, which is 600 to 900 nm. Additionally, the studies used the transcranial light application mode, in which light is applied directly to the head area. The following section aims to provide a comprehensive summary of key findings and limitations of the selected studies.

In an in vivo study conducted by Oxana et al. [7], PBM was administered in combination with 5-aminolevulinic acid (5-ALA) daily for one week. 5-ALA is an acid that has been demonstrated to reduce meningeal lymphatic vessels (MLVs), which are responsible for the suppression of beta-amyloid elimination [7]. PBM was applied fifteen minutes after 5-ALA, with a wavelength of 635 nm, a light dose of 15 J/cm^2^, and a maximum light power of 1 W. Their data demonstrated a notable enhancement in efficacy during the mice’s sleep period, underscoring the therapeutic potential of PBM in addressing AD pathology. The hypothesis posits that the activation of brain lymphatics during deep sleep may be the underlying reason for the heightened efficacy of PBM during this phase [7]. However, a critical concern is that, although the 5-ALA has been shown to be effective in rodents, this may not translate directly to humans. Its ability to reduce MLVs may vary significantly between species due to physiological differences. Cho et al. [9] applied a behavioral approach to study the impact of PBM on the progression of AD. The transgenic 5XFAD mice were subjected to three 20 min sessions of PBM per week for a period of 14 weeks. The light source administered had a peak wavelength of 610 nm, a power intensity of 1.7 mW/cm^2^, and an energy density of 2 J/cm^2^. All mice were subjected to the Morris water maze, passive avoidance, and elevated plus maze tests. The behavioral tests revealed enhanced memory and learning abilities in the early PBM-treated group (from two months of age). Immunohistochemical analysis revealed a notable reduction in beta-amyloid plaques, a primary contributor to AD pathogenesis, along with a decline in the number of neurons undergoing degeneration. The results of both preclinical studies indicate the potential of PBM in reducing the key pathological features of AD. Purushothuman et al. [44] explored the neuroprotective effects of NIR light therapy on two transgenic mouse models of AD, each engineered to develop key AD-related pathologies. The mice were treated with NIR over a period of 4 weeks, and the results showed that in the K3 mice, NIR reduced hyperphosphorylated tau, neurofibrillary tangles, and oxidative stress markers, and restored mitochondrial function in the neocortex and hippocampus. In the *APP*/*PS1* mice, NIR treatment resulted in a notable reduction in the size and number of amyloid-β plaques within the same brain regions. These findings suggest that NIR could be a promising, minimally invasive intervention used to slow or potentially reverse cerebral degeneration associated with AD. One limitation of the above studies involves the distance of light penetration. In small animal models, brain structures are more accessible due to their thinner skull and smaller size. However, when translating to humans, the penetration depth of PBM must be carefully considered, as it may be less effective in reaching deeper brain tissues.

Studies also found an effective reduction in beta-amyloid plaques by using PBM. Tao et al. [45] discovered that 1070 nm with a 10 Hz pulse application in *APP*/*PS1* AD mice models triggered the activation of microglia and cleared out a significant amount of cerebral beta-amyloid plaques. The breakdown of these plaques improved memory and cognitive function in early pathological-stage mice. The average irradiance was reported to be 25 mW/cm^2^ and the treatment length was 60 consecutive days. The results from a behavioral analysis test and the immunofluorescence analysis confirmed the effectiveness of laser therapy in ameliorating AD symptoms. The effectiveness of PBM in removing beta-amyloid plaques from the hippocampus was further investigated by Lu et al. [46] In this study, the experimental cohort of rats received beta-amyloid infusions and were treated with a continuous-wave transcranial diode laser at 808 nm. The treatment was administered for 2 min every day for five consecutive days. The PBM treatment had an irradiance of 25 mW/cm^2^. Histological and immunofluorescence staining results demonstrated that laser treatment decreases oxidative damage and suppresses tau tangles and beta-amyloid plaques.

### 3.2. Clinical Studies of PBM Employed for Curing AD

Due to the lack of definitive knowledge on PBM treatment, there have not been many clinical studies investigating its effects on individuals with AD. However, a few studies have explored its potential in small-scale research efforts.

In a clinical study conducted by Linda Chao [42], eight patients with dementia underwent a 12-week regimen of transcranial and intranasal PBM therapy using an at-home device emitting 810 nm near-infrared light. The participants were randomly assigned to either the control group or the PBM treatment group, with the PBM group receiving therapy three times per week. The study reported significant cognitive improvements in the PBM group, as assessed by the Alzheimer’s Disease Assessment Scale-cognitive subscale (ADAS-cog) and the Neuropsychiatric Inventory (NPI). Additionally, brain imaging revealed enhanced cerebral perfusion and augmented synaptic connectivity within the default-mode network, particularly between the posterior cingulate cortex and lateral parietal nodes. Furthermore, patients exhibited enhanced functional abilities, improved sleep quality, and a reduction in negative emotional outbursts. It is noteworthy that no adverse effects were observed during the course of the study, which indicates that PBM therapy is a well-tolerated, non-invasive home treatment option for dementia. However, the study’s conclusions are limited by the small sample size and the absence of a control group which would have received a sham intervention.

In another clinical study conducted by Berman et al. [43], 11 patients were administered 1070 nm PBM treatment for 6 min per day over a period of 28 days. The patients showed improvements in executive functions, memory recall, and EEG amplitude measurements. The brief duration of the study and the relatively small number of patients were identified as the primary limitations of this study. The results of both clinical studies indicate the potential efficacy of PBM as a therapy for managing Alzheimer’s disease pathology. However, large-scale, long-term studies are keenly needed to validate these findings.

### 3.3. Challenges and Risks of PBM

The use of PBM still cannot yet be considered part of the standard treatment of neuronal disorders, due to lack of knowledge as to the mechanism of PBM on cells and tissues.

Although there is a list of enumerated potential benefits associated with PBM, there are also several risks and challenges that must be addressed in future studies. The main reason for the variability and inconsistency in PBM studies is the lack of standard treatment protocols. The absence of a standard procedure in PBM research complicates comparisons between studies and contributes to inconsistent results.

Another complication associated with PBM is the lack of clinical research and evidence. This problem arises from the difficulty of translating animal studies into clinical practices. There are a multitude of reasons behind this impediment. The human brain structure makes it more difficult to predict how PBM will affect deeper and more distributed brain regions in humans. Additionally, the transgenic animal models do not fully mimic the complex, multifactorial nature of disease in humans.

PBM uses light with many different parameters, such as treatment duration, wavelength, mode of delivery, fluence, and irradiance, which are not consistently applied across different studies. This variability contributes to complications in determining the most effective treatment protocol. Parameters that are effective in mice may need significant adjustment to achieve similar results in humans. More research is needed to establish standard dosimetry guidelines. Without a standard protocol, it will be challenging to integrate PBM into mainstream clinical practice [47]. For long-term treatment in AD with PBM, it is essential to consider the potential risks and side effects, although PBM uses low-level light. High-frequency exposure could potentially lead to the overheating of tissues. This could result in localized thermal damage to brain tissue; solving this problem is of great significance for our research on the therapeutic effects of PBM on brain diseases, such as epilepsy, and might also provide us with new ideas. Also, its long-term effects remain under-researched, particularly factors regarding its impact on neuronal health over years of use. The therapy could induce oxidative stress if the dose is not correctly determined, as excessive mitochondrial activation could lead to overproduction of ROS.

## 4. Various Parameters of Light in PBM for AD Therapy

The efficacy of PBM is contingent upon the specific parameters employed, including the light source, wavelength, power density (irradiance), delivery method, and energy density (fluence). The selection of different values for these parameters has a significant influence on the success of PBM. It has been demonstrated that PBM treatment is biphasic, which indicates that the selection of parameters that fall below or above the optimal range can result in suboptimal, or even adverse, treatment outcomes. The current theories regarding this phenomenon posit that excessive light interaction causes a transient increase in the mitochondrial membrane potential, one which is not sustained as it declines to the baseline level or an even lower potential. Consequently, ATP reserves are depleted, ROS production is increased, and cellular function is compromised. If irradiation persists, cytotoxic pathways are activated, and the metabolic rate is further reduced, ultimately resulting in cellular death. This occurrence is commonly known as the Arndt–Schultz law, which states that any substance which stimulates physiological processes at low doses inhibits them in moderate doses and is lethal in strong doses.

Irradiance and fluence are two important measures of power per unit of area. Irradiance refers to the optical power through a surface area and is expressed as watts/cm^2^. Fluence, however, is defined as the energy contained in a single pulse of light and is expressed as joules/cm^2^. These two parameters have proved to be crucial in determining the success and effectiveness of PBM in various studies.

In an in vitro study conducted by Huang et al. [48], cortical neuronal cells exposed to oxidative stress were continuously irradiated with an 810 nm diode laser for 150 s. The power density was 20 mW/cm^2^, with a total fluence of 3 J/cm^2^, and a spot-size diameter of 5 cm. Through fluorescence staining, the researchers discovered that PBM successfully reduced oxidative cells in the neurons and increased neuronal viability.

In the research study conducted by Sharma et al. [49], neurons were treated with an 810 nm laser with an irradiance of 25 mW/cm^2^ and varying fluences of 0.03, 0.3, 3, 10, and 30 J/cm^2^. The ROS and NO levels were measured using fluorescent probes. The results of this study demonstrated that lower fluences significantly increased ATP and MMP levels, whereas higher fluences caused a decrease in both ATP and MMP levels.

The wavelength of light is another crucial factor in the use of PBM for the treatment of AD. The capacity of light of varying wavelengths to penetrate brain tissue and interact with cellular components is dependent on the wavelength. The variability in wavelength affects the pathways through which cells are stimulated, thereby influencing the therapeutic outcomes. It is therefore of the utmost importance to select an appropriate wavelength for PBM in order to guarantee the success of the AD treatment [50].

In a small pilot double-blind, placebo-controlled trial in humans, Berman et al. [43] utilized 1060–1080 nm light-emitting diodes for transcranial stimulation over 28 sessions. The trial’s results demonstrated improvements in cognitive functions, including executive functioning, memory, and visual attention, as well as trends towards enhanced electroencephalogram (EEG) measures and increased neuroplasticity.

The objective of the study conducted by Sommer et al. [51] was to explore the potential relevance of in vitro treatments for AD. The researchers investigated the effects of 670 nm laser light and epigallocatechin gallate (EGCG) on human neuroblastoma cells which had been laden with amyloid-beta (Aβ42) aggregates. The findings indicated that both 670 nm light and EGCG were effective in reducing Aβ42 aggregates. Additionally, laser irradiation was observed to enhance ATP levels in non-aggregated cells.

In a study conducted by Duan et al. [52], the impact of light-emitting diode (LED) irradiation on Aβ-induced apoptosis, a key contributor to AD, was assessed. The study employed PC12 cell cultures to investigate the impact of LED irradiation at a power of 0.9 W/m^2^ for 60 min on Aβ-induced apoptosis. The findings revealed that LED irradiation significantly reduced Aβ-induced apoptosis within 24 h. A variety of applications of NIR light with different wavelengths have been documented in both in vitro and in vivo studies. LLLT was found to upregulate brain-derived neurotrophic factor (BDNF) in Aβ-treated hippocampal neurons, enhancing neuronal survival and dendrite growth. Another study [53] focused on the effects of 632.8 nm Helium–Neon laser light on oxidative stress and inflammation in astrocytes exposed to oligomeric Aβ, examining these key processes in the Alzheimer’s disease progression. The laser treatment was shown to inhibit Aβ-induced superoxide production, the assembly of NADPH oxidase complexes, and the expression of pro-inflammatory factors, including IL-1β and iNOS.

The efficacy of PBM in vivo is contingent upon the capacity of light to penetrate animal tissues, particularly when targeting deeper brain regions affected by neurodegenerative conditions such as Alzheimer’s disease. A number of studies have indicated that the optimal wavelength for AD treatment in humans falls within the range of 800 to 900 nm. The irradiation value required for effective treatment to the scalp is generally estimated to be up to 60 J/cm^2^, with a power density of less than 250 mW/cm^2^ used to avoid thermal injury [54]. Furthermore, researchers have underscored the pivotal function of light intensity in the process of tissue regeneration [55]. It is therefore imperative to identify the optimal wavelength and power settings for PBM in order to maximize tissue penetration and therapeutic efficacy in the treatment of Alzheimer’s disease.

## 5. Conclusions

Preliminary evidence suggests that PBM therapy may offer a promising non-invasive treatment for AD. Potential benefits include neuroprotection, cognitive function improvement, and an enhanced overall quality of life for patients. PBM represents a promising, non-invasive approach to treating AD, given its wide range of beneficial effects on the brain and its lack of major side effects. A number of studies have indicated that PBM can significantly improve a number of aspects of AD neuropathology, including amyloid-beta and tau pathology, synaptic loss, inflammation, and cognitive impairment. Although the therapy is generally safe, and associated with only mild and transient side effects, such as headaches or scalp itching, it appears that long-term or even indefinite treatment may be necessary to maintain the benefits, as discontinuation or unrelated health issues can lead to regression.

A comprehensive grasp of the mechanisms by which PBM operates is vital for the effective implementation of this approach in the treatment of AD. Although PBM has demonstrated potential in modulating cellular functions, reducing neuroinflammation, and promoting neuroprotection, the precise biological processes it influences in the context of AD remain incompletely understood. A deeper understanding of the manner in which PBM interacts with the complex pathophysiology of AD could not only enhance the efficacy of this therapy but also contribute to a broader understanding of the disease itself. By elucidating the mechanisms through which PBM exerts its effects, we can refine therapeutic strategies, potentially unlocking new avenues for intervention and improving outcomes for patients with AD.

While there is encouraging evidence, the current data on the effectiveness of PBM in AD are still limited and primarily derived from preliminary studies. In order for PBM to be validated as a mainstream treatment, it is essential that robust, randomized, double-blind clinical trials be conducted. These trials will be instrumental in providing the high-quality evidence required to confirm the efficacy and safety of PBM. Given the complex and multifaceted nature of AD, integrating PBM with other therapeutic strategies and refining treatment protocols could potentially offer new and effective options for managing the disease. The transition from experimental studies to clinical practice represents a crucial subsequent phase in the realization of PBM’s full potential for the treatment of AD.

## References

[1] Brown C.S., Ning X., Money A., Alford M., Pan Y., Miller M., Lohman M. (2024). Trends in cause-specific mortality among persons with Alzheimer’s disease in South Carolina: 2014 to 2019. Front. Aging Neurosci..

[2] Tian Z., Wang P., Huang K., Yu J., Zhang M., Liu Y., Zhao H., Zhu B., Huang X., Tong Z. (2023). Photobiomodulation for Alzheimer’s disease: Photoelectric coupling effect on attenuating Aβ neurotoxicity. Lasers Med. Sci..

[3] Conti Filho C.E., Loss L.B., Marcolongo-Pereira C., Rossoni Junior J.V., Barcelos R.M., Chiarelli-Neto O., Silva B.S.D., Passamani Ambrosio R., Castro F.C.D.A.Q., Teixeira S.F. (2023). Advances in Alzheimer’s disease’s pharmacological treatment. Front. Pharmacol..

[4] Maksimovich I.V. (2023). Various Methods of Laser Photobiomodulation Therapy for Alzheimer’s Disease. Med. Res. Arch..

[5] Shen Q., Guo H., Yan Y. (2024). Photobiomodulation for Neurodegenerative Diseases: A Scoping Review. Int. J. Mol. Sci..

[6] Huang Z., Hamblin M.R., Zhang Q. (2024). Photobiomodulation in experimental models of Alzheimer’s disease: State-of-the-art and translational perspectives. Alzheimer’s Res. Ther..

[7] Oxana S.-G., Alexander S., Inna B., Ivan F., Andrey T., Alexander D., Maria T., Daria E., Viktoria A., Arina E. (2023). Mechanisms of phototherapy of Alzheimer’s disease during sleep and wakefulness: The role of the meningeal lymphatics. Front. Optoelectron..

[8] Nagele R.G., Wegiel J., Venkataraman V., Imaki H., Wang K.C., Wegiel J. (2004). Contribution of glial cells to the development of amyloid plaques in Alzheimer’s disease. Neurobiol. Aging.

[9] Cho G.M., Lee S.-Y., Park J.H., Kim M.J., Park K.-J., Choi B.T., Shin Y.-I., Kim N.G., Shin H.K. (2018). Photobiomodulation Using a Low-Level Light-Emitting Diode Improves Cognitive Dysfunction in the 5XFAD Mouse Model of Alzheimer’s Disease. J. Gerontol. Ser. A.

[10] Yang L., Youngblood H., Wu C., Zhang Q. (2020). Mitochondria as a target for neuroprotection: Role of methylene blue and photobiomodulation. Transl. Neurodegener..

[11] Bathini M., Raghushaker C.R., Mahato K.K. (2022). The Molecular Mechanisms of Action of Photobiomodulation Against Neurodegenerative Diseases: A Systematic Review. Cell. Mol. Neurobiol..

[12] Hong N. (2019). Photobiomodulation as a treatment for neurodegenerative disorders: Current and future trends. Biomed. Eng. Lett..

[13] López O.L., DeKosky S.T. (2008). Clinical symptoms in Alzheimer’s disease. Handbook of Clinical Neurology.

[14] Ailioaie L.M., Ailioaie C., Litscher G. (2023). Photobiomodulation in Alzheimer’s Disease—A Complementary Method to State-of-the-Art Pharmaceutical Formulations and Nanomedicine?. Pharmaceutics.

[15] Coronel R., Bernabeu-Zornoza A., Palmer C., Muñiz-Moreno M., Zambrano A., Cano E., Liste I. (2018). Role of Amyloid Precursor Protein (APP) and Its Derivatives in the Biology and Cell Fate Specification of Neural Stem Cells. Mol. Neurobiol..

[16] O’Brien R.J., Wong P.C. (2011). Amyloid precursor protein processing and Alzheimer’s disease. Annu. Rev. Neurosci..

[17] Kang J., Lemaire H.G., Unterbeck A., Salbaum J.M., Masters C.L., Grzeschik K.H., Multhaup G., Beyreuther K., Müller-Hill B. (1987). The precursor of Alzheimer’s disease amyloid A4 protein resembles a cell-surface receptor. Nature.

[18] Medeiros R., Baglietto-Vargas D., LaFerla F.M. (2011). The Role of Tau in Alzheimer’s Disease and Related Disorders. CNS Neurosci. Ther..

[19] Barbier P., Zejneli O., Martinho M., Lasorsa A., Belle V., Smet-Nocca C., Tsvetkov P.O., Devred F., Landrieu I. (2019). Role of Tau as a Microtubule-Associated Protein: Structural and Functional Aspects. Front. Aging Neurosci..

[20] Wang J.-Z., Liu F. (2008). Microtubule-associated protein tau in development, degeneration and protection of neurons. Prog. Neurobiol..

[21] Zhang H., Cao Y., Ma L., Wei Y., Li H. (2021). Possible Mechanisms of Tau Spread and Toxicity in Alzheimer’s Disease. Front. Cell Dev. Biol..

[22] Hamblin M.R. (2018). Mechanisms and Mitochondrial Redox Signaling in Photobiomodulation. Photochem. Photobiol..

[23] Kumar Rajendran N., George B.P., Chandran R., Tynga I.M., Houreld N., Abrahamse H. (2019). The Influence of Light on Reactive Oxygen Species and NF-κB in Disease Progression. Antioxidants.

[24] Dompe C., Moncrieff L., Matys J., Grzech-Leśniak K., Kocherova I., Bryja A., Bruska M., Dominiak M., Mozdziak P., Skiba T.H.I. (2020). Photobiomodulation—Underlying Mechanism and Clinical Applications. J. Clin. Med..

[25] Heinig N., Schumann U., Calzia D., Panfoli I., Ader M., Schmidt M.H.H., Funk R.H.W., Roehlecke C. (2020). Photobiomodulation Mediates Neuroprotection against Blue Light Induced Retinal Photoreceptor Degeneration. Int. J. Mol. Sci..

[26] Li Y., Park J.S., Deng J.H., Bai Y. (2006). Cytochrome c oxidase subunit IV is essential for assembly and respiratory function of the enzyme complex. J. Bioenerg. Biomembr..

[27] Bonora M., Patergnani S., Rimessi A., De Marchi E., Suski J.M., Bononi A., Giorgi C., Marchi S., Missiroli S., Poletti F. (2012). ATP synthesis and storage. Purinergic Signal..

[28] Pastore D., Greco M., Passarella S. (2000). Specific helium-neon laser sensitivity of the purified cytochrome c oxidase. Int. J. Radiat. Biol..

[29] de Freitas L.F., Hamblin M.R. (2016). Proposed Mechanisms of Photobiomodulation or Low-Level Light Therapy. IEEE J. Sel. Top. Quantum Electron..

[30] Karu T.I. (2010). Multiple roles of cytochrome c oxidase in mammalian cells under action of red and IR-A radiation. IUBMB Life.

[31] Farivar S., Malekshahabi T., Shiari R. (2014). Biological effects of low level laser therapy. J. Lasers Med. Sci..

[32] Lima P.L.V., Pereira C.V., Nissanka N., Arguello T., Gavini G., Maranduba C.M.d.C., Diaz F., Moraes C.T. (2019). Photobiomodulation enhancement of cell proliferation at 660 nm does not require cytochrome c oxidase. J. Photochem. Photobiol. B Biol..

[33] Wang X., Wang W., Li L., Perry G., Lee H.G., Zhu X. (2014). Oxidative stress and mitochondrial dysfunction in Alzheimer’s disease. Biochim. Biophys. Acta.

[34] Zong Y., Li H., Liao P., Chen L., Pan Y., Zheng Y., Zhang C., Liu D., Zheng M., Gao J. (2024). Mitochondrial dysfunction: Mechanisms and advances in therapy. Signal. Transduct. Target. Ther..

[35] Zorov D.B., Juhaszova M., Sollott S.J. (2014). Mitochondrial reactive oxygen species (ROS) and ROS-induced ROS release. Physiol. Rev..

[36] Petronilli V., Szabò I., Zoratti M. (1989). The inner mitochondrial membrane contains ion-conducting channels similar to those found in bacteria. FEBS Lett..

[37] Zhang L., Zhang Y., Xing D. (2010). LPLI inhibits apoptosis upstream of Bax translocation via a GSK-3beta-inactivation mechanism. J. Cell Physiol..

[38] McAllister A.K., Lo D.C., Katz L.C. (1995). Neurotrophins regulate dendritic growth in developing visual cortex. Neuron.

[39] Schwartz P.M., Borghesani P.R., Levy R.L., Pomeroy S.L., Segal R.A. (1997). Abnormal cerebellar development and foliation in *BDNF*^−/−^ mice reveals a role for neurotrophins in CNS patterning. Neuron.

[40] Mertz K., Koscheck T., Schilling K. (2000). Brain-derived neurotrophic factor modulates dendritic morphology of cerebellar basket and stellate cells: An in vitro study. Neuroscience.

[41] Sperandio F.F., Giudice F.S., Corrêa L., Pinto D.S., Hamblin M.R., de Sousa S.C. (2013). Low-level laser therapy can produce increased aggressiveness of dysplastic and oral cancer cell lines by modulation of Akt/mTOR signaling pathway. J. Biophotonics.

[42] Chao L.L. (2019). Effects of Home Photobiomodulation Treatments on Cognitive and Behavioral Function, Cerebral Perfusion, and Resting-State Functional Connectivity in Patients with Dementia: A Pilot Trial. Photobiomodul. Photomed. Laser Surg..

[43] Berman M.H., Halper J.P., Nichols T.W., Jarrett H., Lundy A., Huang J.H. (2017). Photobiomodulation with Near Infrared Light Helmet in a Pilot, Placebo Controlled Clinical Trial in Dementia Patients Testing Memory and Cognition. J. Neurol. Neurosci..

[44] Purushothuman S., Johnstone D.M., Nandasena C., Mitrofanis J., Stone J. (2014). Photobiomodulation with near infrared light mitigates Alzheimer’s disease-related pathology in cerebral cortex—Evidence from two transgenic mouse models. Alzheimer’s Res. Ther..

[45] Tao L., Liu Q., Zhang F., Fu Y., Zhu X., Weng X., Han H., Huang Y., Suo Y., Chen L. (2021). Microglia modulation with 1070-nm light attenuates Aβ burden and cognitive impairment in Alzheimer’s disease mouse model. Light Sci. Appl..

[46] Lu Y., Wang R., Dong Y., Tucker D., Zhao N., Ahmed M.E., Zhu L., Liu T.C., Cohen R.M., Zhang Q. (2017). Low-level laser therapy for beta amyloid toxicity in rat hippocampus. Neurobiol. Aging.

[47] Zhang R., Qu J. (2023). The Mechanisms and Efficacy of Photobiomodulation Therapy for Arthritis: A Comprehensive Review. Int. J. Mol. Sci..

[48] Huang Y.Y., Nagata K., Tedford C.E., McCarthy T., Hamblin M.R. (2013). Low-level laser therapy (LLLT) reduces oxidative stress in primary cortical neurons in vitro. J. Biophotonics.

[49] Sharma S.K., Kharkwal G.B., Sajo M., Huang Y.Y., De Taboada L., McCarthy T., Hamblin M.R. (2011). Dose response effects of 810 nm laser light on mouse primary cortical neurons. Lasers Surg. Med..

[50] Zein R., Selting W., Hamblin M.R. (2018). Review of light parameters and photobiomodulation efficacy: Dive into complexity. J. Biomed. Opt..

[51] Sommer A.P., Bieschke J., Friedrich R.P., Zhu D., Wanker E.E., Fecht H.J., Mereles D., Hunstein W. (2012). 670 nm Laser Light and EGCG Complementarily Reduce Amyloid-β Aggregates in Human Neuroblastoma Cells: Basis for Treatment of Alzheimer’s Disease?. Photomed. Laser Surg..

[52] Duan R., Zhu L., Liu T.C.-Y., Li Y., Liu J., Jiao J., Xu X., Yao L., Liu S. (2003). Light emitting diode irradiation protect against the amyloid beta 25–35 induced apoptosis of PC12 cell in vitro. Lasers Surg. Med..

[53] Yang X., Askarova S., Sheng W., Chen J.K., Sun A.Y., Sun G.Y., Yao G., Lee J.C.M. (2010). Low energy laser light (632.8 nm) suppresses amyloid-β peptide-induced oxidative and inflammatory responses in astrocytes. Neuroscience.

[54] Enengl J., Hamblin M.R., Dungel P. (2020). Photobiomodulation for Alzheimer’s Disease: Translating Basic Research to Clinical Application. J. Alzheimers Dis..

[55] Wang P., Li T. (2019). Which wavelength is optimal for transcranial low-level laser stimulation?. J. Biophotonics.

